# The Photoluminescence and Vibrational Properties of Black Phosphorous Sheets Chemically/Electrochemically Functionalized in the Presence of Diphenylamine

**DOI:** 10.3390/polym14214479

**Published:** 2022-10-22

**Authors:** Mihaela Baibarac, Teodora Burlanescu, Malvina Stroe, Ion Smaranda, Catalin Negrila

**Affiliations:** National Institute of Materials Physics, R077125 Bucharest, Romania

**Keywords:** diphenylamine, black phosphorus, Raman scattering, IR spectroscopy, photoluminescence

## Abstract

In this work, new information concerning the optical properties of black phosphorus (BP) sheets chemically/electrochemically functionalized with diphenyl amine (DPA) and its macromolecular compound (poly(diphenylamine) (PDPA)) in the absence/presence of phosphotungstic acid (PTA) is reported. Raman scattering and FTIR spectroscopy studies indicate that the interaction of BP with PTA leads to the elimination of the P_x_O_y_ layer onto the surface of the BP sheets. In the case of the chemical interaction of BP with DPA, the reaction product corresponds to DPA chemically functionalized BP sheets having an imino-phosphorane (IP) structure. The electrochemical oxidation of BP sheets chemically functionalized with DPA in the presence of PTA leads to an increase in the weight of P-N bonds as a consequence of the generation of PDPA doped with the PTA heteropolyanions, as shown by FTIR spectroscopy and Raman scattering. This process is evidenced by a shift of the Raman line from 362 cm^−1^ to 378 cm^−1^, assigned to the A_1g_ mode. This change was explained by taking into account the compression of the layers containing P atoms, which is induced by PDPA macromolecular chains. The decrease in the intensity of the PL spectra of DPA as well as PDPA, in the presence of BP, indicates that BP acts as a PL quenching agent for these compounds. A preferential orientation of the PDPA doped with the PTA heteropolyanions on the surface of BP sheets is highlighted by the variation of the binding angle of the PDPA on the surface of BP sheets from 44.7° to 39.9°.

## 1. Introduction

Phosphorene (P) has been receiving the attention of researchers since 2014 [1]. The main methods of synthesis of P are (i) the mechanical cleavage of black phosphorus (BP) [1]; (ii) exfoliation by ultrasonic processes in the liquid phase [2,3]; (iii) plasma-assisted synthesis [4]; and (iv) the passivation of BP with Nafion [5]. An issue with using BP mono- and few-layer nanosheets is their instability in ambient oxygen, leading to oxidative degradation, as reported in [6,7,8,9,10]. In order to avoid this problem, two functionalization processes are often used, i.e., non-covalent functionalization with electron-withdrawing molecules [11], such as ionic liquids [12,13] or 2D sheet polymers [14], and covalent functionalization with monovalent hydrogen- or fluorine-type addends when in the P lattice, with sp^3^-hybridization occurring in regions in which P atoms have undergone sp^2^-hybridization [14]. The addition of the electrophiles carbenes, oxygen, Lewis acid or nitrenes to BP has allowed the generation of the addition compounds phosphonium ylid, phosphinoxide, Lewis acid adducts and imino-phosphorane [14].

A sustained effort has been made in recent years to prepare new composite materials with both insulating polymers (e.g., polystyrene [15]) and conducting polymers (e.g., poly (3,4-ethylenedioxythiophene) [16]) as the host matrix and guest structures of BP or phosphorene (P), which are reported to have a high stability. Other composites based on P and conjugated polymers that have attracted attention have been poly (3-hexyl thiophene) [17] and polyaniline [18]. Recent studies have demonstrated that the interaction of BP with PANI leads to p-π conjugated structures and π-π heterostructures [18]. Despite these efforts, a less studied aspect is the interaction of the monomers of conjugated polymers with BP sheets [19]. In order to exemplify the importance of knowing the nature of the interactions between BP sheets and monomers, in this paper new information on the interaction of BP sheets with poly(diphenylamine) (PDPA) monomers is reported.

The first study looking at the interaction of BP with compounds having amine functional groups was reported by S. Li et al., who investigated BP surface functionalization with amines (BP-NH_2_) (secondary alkylamine (diethylenetriamine), primary arylamine (p-phenylenediamine) and cyclamine (4-amino-2,2,6,6-tetramethylpiperidine)) using FTIR spectroscopy, Raman scattering and X-ray photoelectron spectroscopy (XPS) [20]. In contrast with Ref. [20], the attention in this work will be focused on the interaction of the BP sheets with the secondary arylamine diphenylamine (DPA). According to the studies presented below, the BP sheets functionalized with DPA will be used to obtain new BP-functionalized composites with the polymer derived from DPA, i.e., PDPA. According to the studies presented below, the BP sheets functionalized with DPA will be used to obtain new BP-functionalized composites with the polymer derived from DPA, i.e., PDPA. As shown in this paper, the synthesis method of these can be cyclic voltammetry, involving the electrochemical oxidation of BP sheets chemically functionalized with DPA in the presence of phosphotungstic acid (PTA).

The development of the synthesis methods of P or BP and their composites have allowed new applications to be developed in the fields of: (i) information storage [21]; (ii) delaying flame ignition [22]; (iii) cancer therapy [23]; (iv) rechargeable Li and Na batteries [24,25]; (v) photocatalysis [26]; (vi) gas sensors [27]; and (vii) field effect transistors [28].

Compared to this progress, in this work our attention will be focused on the chemical interaction of BP with DPA and phosphotungstic acid (PTA), as well as the electrochemical oxidation of DPA chemically functionalized BP sheets, highlighting their optical properties.

## 2. Materials and Methods

DPA (≥99%), H_3_PW_12_O_40_ x H_2_O (99.995% trace metals basis, PTA), HCl (37%) and N, N-dimethylformamide (DMF, anhydrous, 99.8%) were purchased from Sigma-Aldrich, St. Louis, MO, USA. BP crystal (99.995%) was purchased from NanoIntegris.

In the case of the chemical interaction of DPA with BP, the concentration of BP in the mass of the DPA/BP mixture prepared in solid state was varied from 0 wt.% to 0.5 wt.% and 1 wt.%.

The chemical interaction of BP with PTA was carried out when the concentration of BP in the mass of the BT/PTA mixture was equal to 1.7 wt.%.

DPA electrochemical oxidation was performed using cyclic voltammetry, with the synthesized solution consisting of 10^−2^ M DPA, 10^−3^ M PTA, and 1M HCl in the semi-aqueous solution of dimethylformamide (DMF) and H_2_O, having a volumetric ratio of 1:1. In order to obtain composites containing BP sheets and macromolecular compounds of DPA, in the above reaction mixture BP sheets (0.1, 0.2 and 0.5 mg/mL) were added. In the absence of BP, the electrochemical oxidation of DPA leads to the formation of a film of poly(diphenylamine) (PDPA) doped with PTA heteropolyanions on the working electrode surface [29]. Before DPA electrochemical oxidation, BP was exfoliated in DMF using an ultrasonication process for 20 min. The potential range used for recording the first five cyclic voltammograms was (+100; +960) mV vs. Ag/AgCl, with the scanning rate of the potential being equal to 50 mV/s. The electrochemical cell was connected to a Voltalab 80 potentiostat/galvanostat from Radiometer Analytical.

The information about the products resulting from the interactions of BP with DPA and PTA, as well as from the electrochemical oxidation of DPA in the presence of BP, PTA and HCl was obtained by Raman scattering and FTIR spectroscopy.

The Raman spectra of BP, DPA, PTA, and the BP sheets chemically/electrochemically functionalized with DPA or their macromolecular compounds were recorded with a FTRaman spectrophotometer, MultiRam model, from Bruker.

The IR spectra of the samples prepared in this work were recorded with an FTIR spectrophotometer, Vertex 80 model, from Bruker (Billerica, MA, USA).

The photoluminescence (PL) spectra of the DPA chemically/electrochemically functionalized BP sheets were recorded with a Fluorolog spectrophotometer (3.2.2.1) from Horiba Jobin Yvon with a right-angle geometry. All PL spectra were recorded at room temperature, having slides of 3 nm both at excitation and emission. For the measurements of the anisotropic PL, before recording the PL spectra in the polarized light of the PDPA electrochemically functionalized BP sheets and PDPA, a checking polarizer alignment was carried out using a Ludox sample.

The X-ray photoelectron spectroscopy (XPS) spectra of PDPA, BP and PDPA electrochemically functionalized BP sheets were recorded with a SPECS spectrometer having a Phoibos 150 electrone energy analyzer, a monochromatic X-ray source of the type AlKa 1486.74 eV (SPECS Surface Nano Analysis GmbH, Berlin, Germany) and Spectral Data Processor SDPv7.0 software (XPS International, Mountain View, CA, USA).

## 3. Results and Discussion

### 3.1. Optical Evidence for the Interaction of BP with PTA and DPA

**Figure 1:**
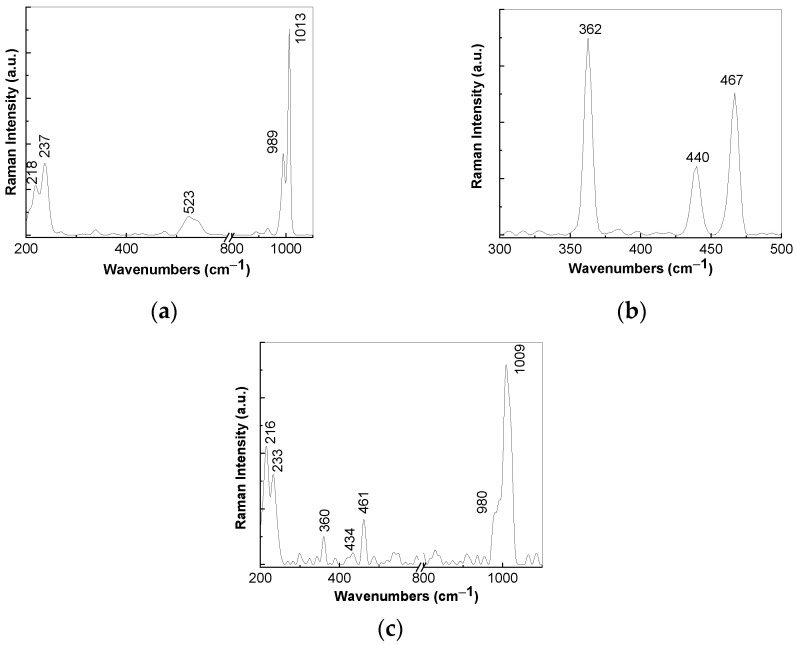
Raman spectra of PTA (**a**), BP (**b**) and BP when interacting with PTA (**c**).

According to Figure 1a, the Raman spectrum of PTA is characterized by an intense line with a maximum at 1013 cm^−^^1^, having a shoulder at 989 cm^−^^1^, which accompanies another two Raman lines of low intensity at 523 cm^−^^1^ and 218–237 cm^−^^1^. The Raman lines of PTA peaked at 1013 cm^−^^1^ and 989 cm^−^^1^ and were assined to the vbirational modes of the P-O bond in the PO_4_ sites and the W=O bond in PTA [30].

**Scheme 1:**
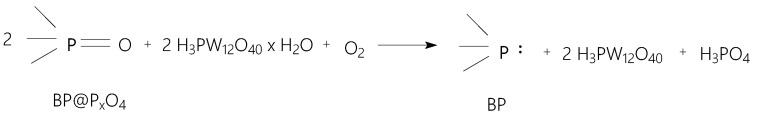
The reaction of BP having a P_x_O_y_ layer (BP@ P_x_O_y_) with H_3_PW_12_O_40_ x H_2_O.

The Raman lines at 523 cm^−1^ and 218-237 cm^−1^ were attributed to the vibrational mode of bending W-O-W and stretching W=O [31]. According to Figure 1b, the Raman spectrum of BP shows three lines with maxima at 362 cm^−1^, 440 cm^−1^ and 467 cm^−1^, which were attributed to the A_1g_, B_2g_ and A_2g_ vibrational modes, respectively [32]. The interaction of BP with PTA led to the following changes shown in Figure 1c: (i) a downshift of the Raman lines of PTA from 1013 cm^−1^ and 989 cm^−1^ (Figure 1a) to 1009 cm^−1^ and 980 cm^−1^, respectively (Figure 1c); (ii) a change in the ratio between the intensities of the Raman lines of PTA in the spectral ranges 200–300 cm^−1^ and 900–1050 cm^−1^ from 0.35 (Figure 1a) to 0.45 (Figure 1c); and (iii) a downshift of the Raman line of BP from 362 cm^−1^, 440 cm^−1^ and 467 cm^−1^ (Figure 1b) to 360 cm^−1^, 434 cm^−1^ and 461 cm^−1^, respectively (Figure 1c), accompanied by a change in the ratio between the intensities of the Raman line of BP, which peaked at 362 cm^−1^ and 467 cm^−1^ from 1.38 (Figure 1b) to 0.63 (Figure 1c). At present, it is well known that the A_1g_/A_2g_ ratio provides information about the oxidation state of BP [33]. The decrease in the A_1g_/A_2g_ ratio up to the value 0.63 is a consequence of the interaction of the oxidized BP from the interface the BP few-layer surface with the water molecules existing in H_3_PW_12_O_40_ x H_2_O. Previous studies have demonstrated that the interaction with the P_x_O_y_ layer at the BP surface involves the transformation of P_x_O_y_ into H_3_PO_4_ and PH_3_ [34]. In our case, Scheme 1 describes the interaction of BP having a P_x_O_y_ layer with H_3_PW_12_O_40_ x H_2_O.

The adsorption of H_3_PW_12_O_40_ onto the BP surfaces induces a red-shift of the Raman lines, assigned to the vibrational modes A_1g_, B_2g_ and A_2g_ with ~2 cm^−1^, 6 cm^−1^ and 6 cm^−1^, respectively. An explanation of this must take into account the fact that there is a hindrance of the oscillations of the P atoms belonging to BP. A consequence of this is the decrease in energy of Raman scattering, which induces the red-shift of the Raman lines of the A_1g_, B_2g_ and A_2g_ vibrational modes.

**Figure 2:**
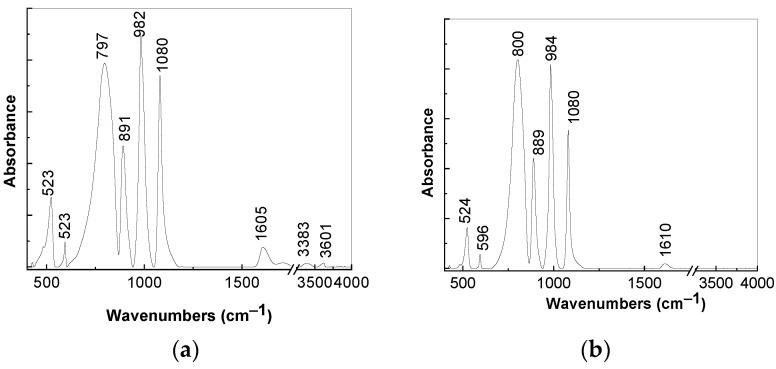
The IR spectra of PTA (**a**) and BP when interacting with PTA (**b**).

Additional information about the reaction of BP having a P_x_O_y_ layer with H_3_PW_12_O_40_ x H_2_O is shown in Figure 2, obtained using FTIR spectroscopy. Figure 2a shows the main IR bands of PTA situated at 523 cm^−1^, 594 cm^−1^, 797 cm^−1^, 891 cm^−1^, 982 cm^−1^, 1080 cm^−1^, 1605 cm^−1^ and 3383–3601 cm^−1^, being assigned to the vibrational modes of the deformation of the O-P-O bond, symmetrical stretching of the W-O-W bond, W-O_c_-W stretching mode (O_c_ corresponds to corner oxygen atoms), W-O_e_-W stretching mode (O_e_ corresponds to edge oxygen atoms), anti-symmetrical stretching of the W=O bond, P-O-W stretching mode, the deformation vibrational mode of water molecules and the stretching mode of hydroxyl groups in water molecules, respectively [35,36].

The interaction of BP having a P_x_O_y_ layer with PTA induces the following changes, shown in Figure 2: (i) a shift of the IR band from 797 cm^−1^ to 800 cm^−1^, simultaneously with an increase in its absorbance; (ii) a change in the ratio between the absorbance of the IR bands, peaking at 982–984 cm^−1^ and 1080 cm^−1^ from 1.21 (Figure 2a) to 1.47 (Figure 2b); (iii) the disappearance of the IR bands at 3383–3601 cm^−1^; and (iv) the decrease in the absorbance of the IR band localized in the spectral range 1550–1650 cm^−1^, the variation of which was accompanied by a shift from 1605 cm^−1^ (Figure 2a) to 1610 cm^−1^ (Figure 2b). These results confirm once more that the changes reported in Figure 1 originate from the interaction of P_x_O_y_ with the water from PTA.

**Figure 3:**
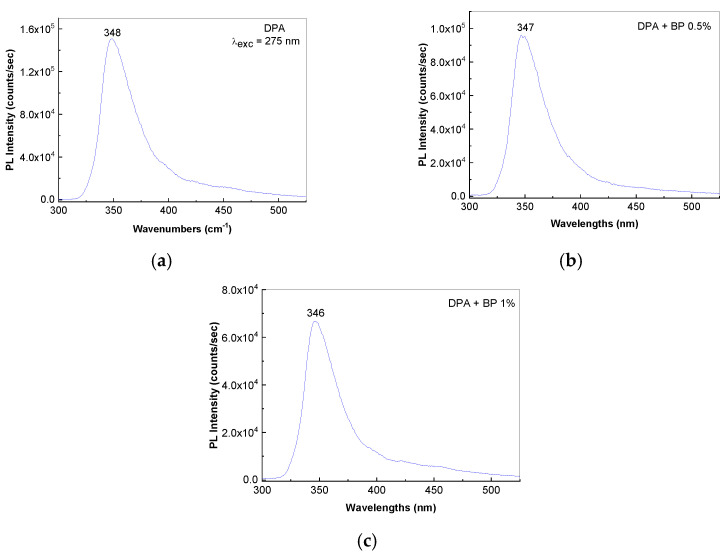
The PL spectra of DPA before (**a**) and after the interaction with BP 0.5 wt.% (**b**) and 1 wt.% (**c**), when the excitation wavelength is 275 nm.

In order to illustrate the interaction of BP with DPA, Figure 3 shows the PL spectra of DPA and the mixture of DPA:BP when the BP concentration is equal to 0.5 wt.%. and 1 wt.%.

**Figure 4:**
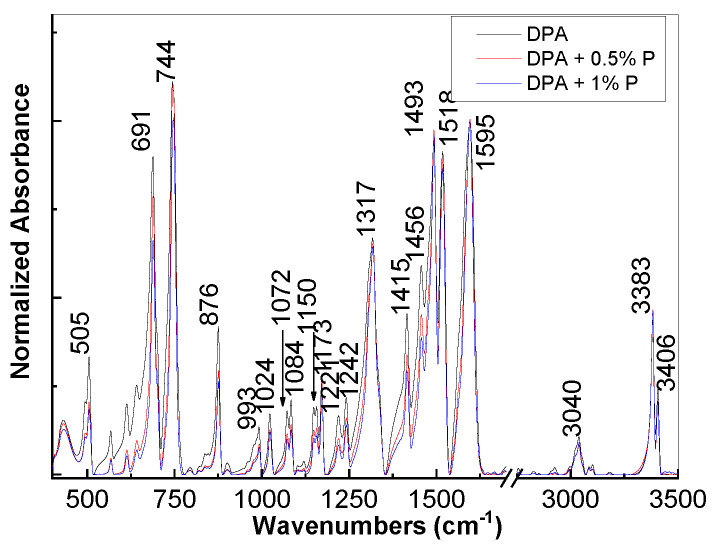
The IR spectra of DPA before (black curve) and after the interaction with BP; the BP concentration in the DPA:BP mixture is equal to 0.5 wt.% (red curve) and 1 wt.% (blue curve).

In all three cases, the PL spectra are characterized by a band with maximums at 348 nm, 347 nm and 346 nm, and with intensities equal to 1.51 × 10^6^ counts/sec (Figure 3a), 9.57 × 10^5^ counts/s (Figure 3b) and 6.68 × 10^4^ counts/s (Figure 3c), respectively. The lower intensity of the PL spectra in Figure 3b,c in comparison with Figure 3a indicates a quenching process of the DPA PL induced by BP sheets. According to Figure 4, the IR spectrum of DPA is characterized by bands with maxima at ~691–744 cm^−1^, 876 cm^−1^, 1173 cm^−1^, 1242 cm^−1^, 1317 cm^−1^, 1415 cm^−1^, 1456–1493–1518 cm^−1^, 1595 cm^−1^ and 3383 cm^−1^, attributed to the vibrational modes: the out-of-plane deformation of the benzene ring, deformation of the benzene ring, C-H bond, stretching of the C-N bond, C-H bond, N-H bond + C-H bond, C-C + C-H + N-H stretching, C-C bond stretching in the benzene ring and N-H bond, respectively [37]. In the presence of BP, gradual decreases in the absorbance of the IR bands localized at 691–744 cm^−1^ and 876 cm^−^^1^ were observed.

**Scheme 2:**
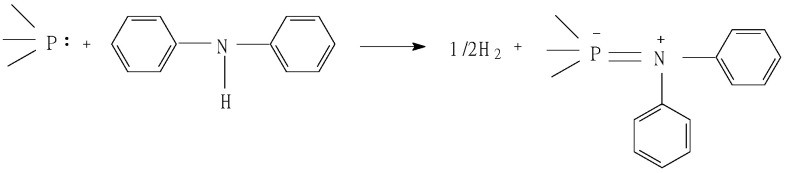
The interaction of DPA with BP.

In our opinion, the decrease in the absorbance of the IR bands localized at 691–744 cm^−1^ and 876 cm^−1^ can be explained by the interaction of BP with DPA, when during the chemical interaction a covalent functionalization of the BP layers with DPA takes place, which leads to an intercalation of DPA between the BP layers, with a part of BP layers thus being exfoliated. The reaction product corresponds to the iminophosphorene-type structure [14], according to Scheme 2.

Summarizing the above results, we conclude that: (i) the interaction of BP@P_x_O_y_ with PTA leads to a decrease in the oxidation state of BP and (ii) the interaction of BP with DPA leads to the generation of the DPA chemically functionalized BP sheets, which have an iminophosphorene-type structure.

### 3.2. Electrochemical Oxidation of the DPA Chemically Functionalized BP Sheets Determined by Correlated Studies of Photoluminescence, Raman Scattering, FTIR Spectroscopy and X-ray Photoelectron Spectroscopy

**Figure 5:**
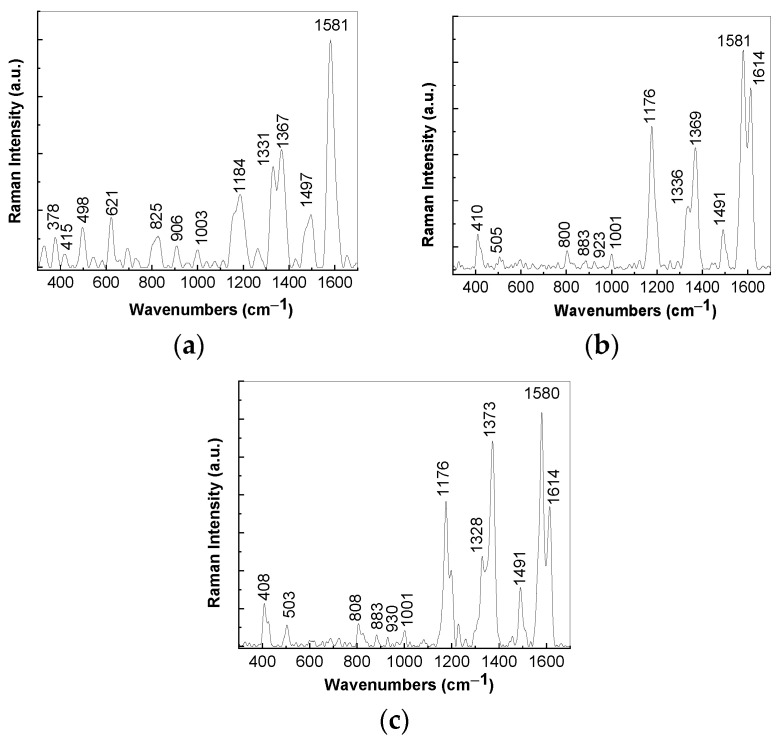
Raman spectra of the composites resulting from the electrochemical oxidation of the DPA chemically functionalized BP sheets when the concentration of BP in DMF in the synthesis mixture is 0.5 mg/mL (**a**), 0.2 mg/mL (**b**) and 0.1 mg/mL (**c**).

In order to show the vibrational properties of the composites resulting from the electrochemical oxidation of the DPA chemically functionalized BP sheets, the studies of Raman scattering and IR spectroscopy are shown here. The main Raman lines observed in Figure 5a–c have maxima at ~415–498 cm^−1^, 621–825 cm^−1^, 906–1003 cm^−1^, 1184 cm^−1^, 1331 cm^−1^, 1367 cm^−1^, 1497 cm^−1^, 1581 cm^−1^ and 1614 cm^−1^, belonging to macromolecular compound of DPA, i.e., PDPA doped with PTA heteropolyanions. These Raman lines are attributed to the vibrational modes caused by the deformation of the benzene ring, out-of-side of the benzene plane, P-O in the PO_4_ structures of the heteropolyanion, of C-H bond in the benzene ring, C _aromatic ring_ - N in the radical cation type structural units N, N′-diphenyl benzidine, C-C stretching in the benzene ring + C-H bond in the benzene ring, C=N stretching, C=C stretching in the quinoid ring and C-C stretching in the benzene ring, respectively [37,38,39,40].

**Figure 6:**
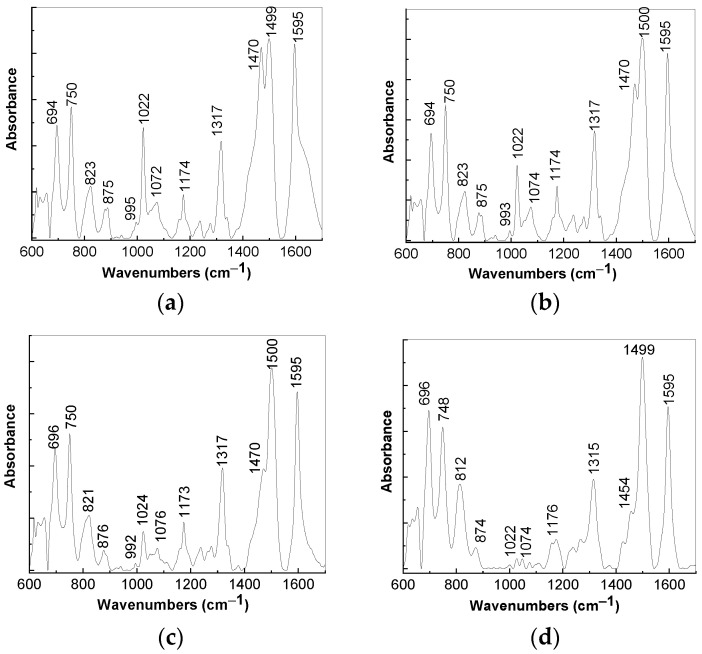
IR spectra of the composite resulting from the electrochemical oxidation of the DPA chemically functionalized BP sheets when the concentration of BP in DMF in the synthesis mixture is 0.5 mg/mL (**a**), 0.2 mg/mL (**b**) and 0.1 mg/mL (**c**). Figure (**d**) shows the IR spectrum of PDPA doped with the PTA heteropolyanions.

The decrease in the weight of the BP sheets in the reaction mixture induces: (i) a shift in the Raman lines from 1184 and 1497 cm^−1^ to 1176 and 1491 cm^−1^, respectively; (ii) an increase in the intensity of the Raman line from 1614 cm^−1^; and (iii) a change in the ratio between the intensities of the Raman lines located in the spectral range 1300–1380 cm^−1^. This fact suggests that: (i) the macromolecular compound contains a higher proportion of quinoid rings, with the Raman spectrum being characterized in the spectral range 1550–1650 cm^−1^, only by the line at 1581 cm^−1^ (Figure 5a), and (ii) the electrochemical oxidation of the DPA chemically functionalized BP sheets induces changes in the vibrational mode of the C=N bonds as a consequence of the reactions at the electrode/electrolyte interface when the C=N bonds are transformed into C-N bonds. This variation can explain the change in the position of the Raman line from 1176 cm^−1^ (Figure 5c) to 1184 cm^−1^ (Figure 5a). In Figure 5a, one can see a Raman line at 378 cm^−1^, which does not belong to DPA or its polymer. A puzzling fact is that a shift of the A_1g_ vibrational mode of BP sheets from 362 cm^−1^ to 378 cm^−1^ was reported when the sheets containing P atoms were compressed [41]. In our case, the variation of the Raman line attributed to the A_1g_ vibrational mode from 362 cm^−1^ to 378 cm^−1^ can be explained by taking into account the compression of the layers containing P atoms, which is induced by PDPA. In Figure 6d, it can be seen that the IR spectrum of PDPA doped with the PTA heteropolyanions is characterized by the IR bands having maxima at 696 cm^−1^, 748 cm^−1^, 812 cm^−1^, 874 cm^−1^, 922 cm^−1^, 1022 cm^−1^, 1074 cm^−1^, 1176 cm^−1^, 1315 cm^−1^, 1454 cm^−1^, 1499 cm^−1^ and 1595 cm^−1^, attributed to the vibrational modes of the inter-ring deformation, ring deformation, W-O_c_-W (O_c_ corresponds to oxygen from the corner of the heteropolyanion structure), W-O_e_-W (O_e_ corresponds to oxygen from the side of the heteropolyanion structure), W=O, the deformation of the quinoid ring, the stretching of the P-O-W bond, the C-H bond in the benzene (B) ring, C _aromatic_ - N, the stretching of the C=N + C-H bond in the benzene ring, the stretching of the C-C bond in the benzene (B) ring + the stretching vibration of the C=C bond in the quinoid ring (Q) and the stretching of the C=C bond in the -NH^+^=Q=Q=NH^+^- structure, respectively [37,38,39].

**Scheme 3:**
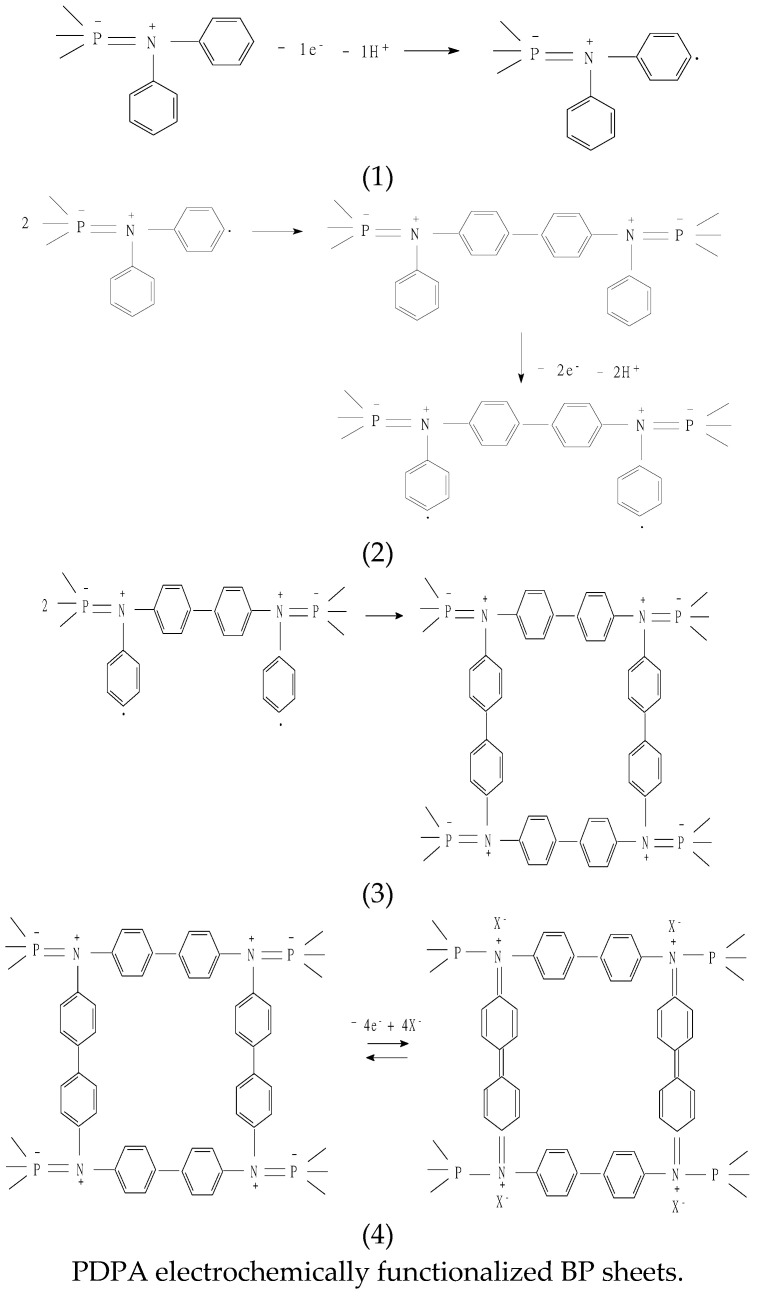
The reactions describing the electrochemical oxidation reactions of the DPA chemically functionalized BP sheets, which leads to composites with BP sheets electrochemically functionalized with macromolecular compounds having repeating units made through doping DPA with PTA heteropolyanions (X^-^ corresponds to the PTA heteropolyanion).

Figure 6a–c highlight the following changes in the case of the composite resulting from the electrochemical oxidation of the DPA chemically functionalized BP sheets: (i) a shift of the maximum of the IR band from 812 cm^−1^ to 823 cm^−1^; (ii) an increase in the absorbance of the IR bands located at 1022 cm^−1^ and 1072–1076 cm^−1^; and (iii) a shift of the IR band from 1454 cm^−1^ to 1470 cm^−1^ simultaneously with the change in the ratio between the absorbances of the IR bands from 1454 to 1470 cm^−1^ and 1499 cm^−1^. These variations can be explained by taking into account the increase in the number of covalent bonds between the BP sheets and DPA-type structural units, when steric hindrance affects both the quinoid rings and the PTA heteropolyanions, which compensates the existing positive charges on the PDPA macromolecular chain. In order to understand the chemical structures of composites resulting from the electrochemical oxidation of DPA chemically functionalized BP sheets, Scheme 3 shows the reactions that allow the macromolecular chain growth, i.e., of dimers, tetramers and PDPA.

According to Scheme 3, reaction (1) leads to a radical of the DPA chemically functionalized BP sheets, which is instable and this will interact with another radical, leading to a dimer (reaction (2)). Next, the oxidation reaction of the dimer will lead to a diradical (reaction (2)) which, being unstable, will interact with another dimer, leading to the compound shown in reaction (3). Reaction (4) shows the oxidation reaction of the tetramer, when a composite of the type of BP layers functionalized with DPA tetramers doped with PTA heteropolyanions results. Further growth of the macromolecular chain through oxidation reactions leads to a composite made of PDPA electrochemically functionalized BP sheets.

**Figure 7:**
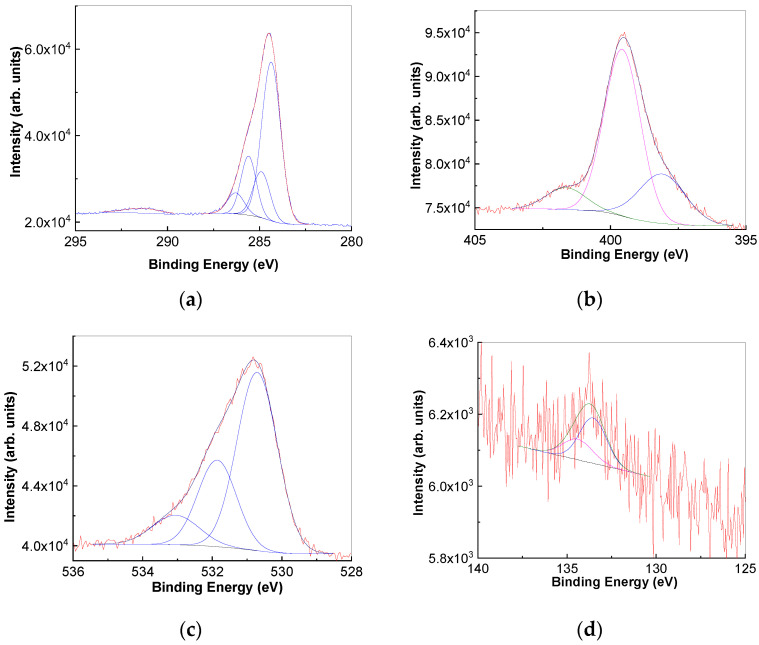
X-ray photoelectron spectroscopy (XPS) C1s (**a**), N1s-Mo3p3/2 (**b**), O1s (**c**) and P2p (**d**) spectra of PDPA doped with TPA heteropolyanions. Red and blue curves correspond to experimental results and their deconvolution.

**Figure 8:**
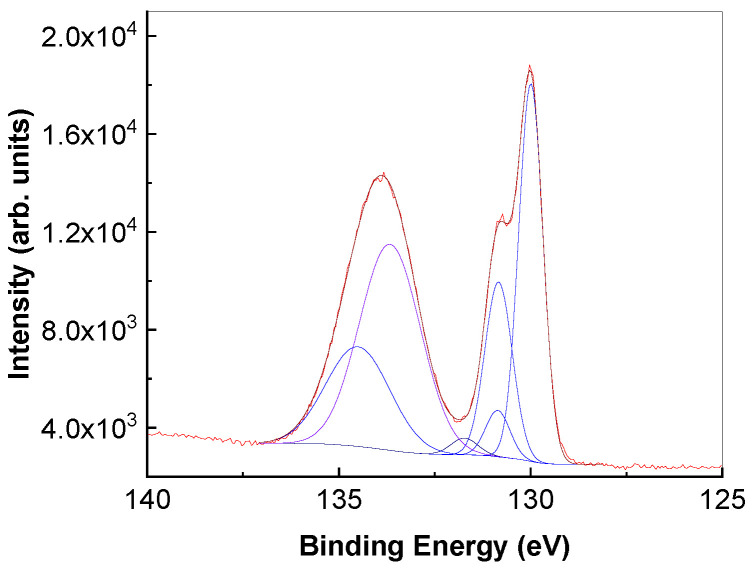
The XPS P2p spectra of BP. Red and blue curves correspond to experimental results and their deconvolution.

**Figure 9:**
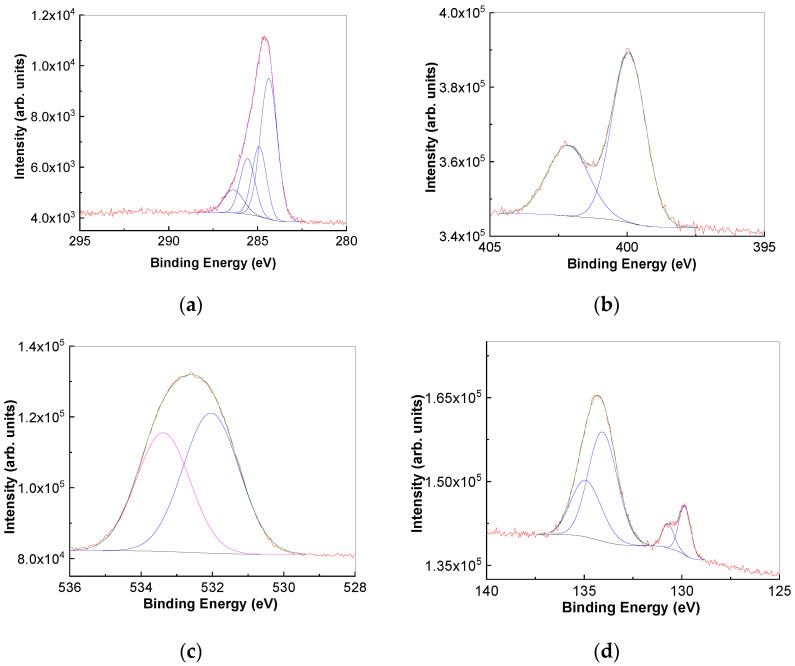
The XPS C1s (**a**), N1s (**b**), O1s (**c**) and P2p (**d**) spectra of PDPA electrochemically functionalized BP sheets. Red and blue curves correspond to experimental results and their deconvolution.

In order to show additional experimental evidence for the PDPA electrochemically functionalized BP sheets, in Figure 7, Figure 8 and Figure 9 the XPS spectra of PDPA, BP and the PDPA electrochemically functionalized BP sheets are shown.

Figure 7 shows the case of PDPA doped with PTA heteropolyanions in: (i) the XPS C1s spectrum, a peak of high intensity at 284.37 eV, accompanied by another four peaks at 284.9 eV, 285.6 eV, 286.3 eV and 291.35 eV, which were assigned to sp^2^ C atoms in the benzene ring, the C-C and C-H bonds, C-N bond, the C-O and C-N+ bonds and π-π* transitions of sp^2^ C atoms [42]; (ii) the XPS N1s-Mo3p^3/2^ spectrum, with three peaks at 398.09 eV, 399.56 eV and 401.65 eV assigned to Mo3 p^3/2^ (where the Mo oxidized state is Mo^6+^), N1s of the amine groups and NH^+^ [43,44]; (iii) the XPS P2p spectrum, with a peak of low intensity corresponding to the TPA heteropolyanions [43,44]; and (iv) XPS O1s, which reveals an intense peak at 530.71 eV accompanied of two peaks at 531.86 eV and 533.05 eV, attributed to the O-Mo bond, adsorbed O and the C-O bonds [43,44].

The deconvolution of Figure 8 illustrates six peaks at 129.99 eV–130.83 eV, 130.86 eV–131.73 eV and 133.67 eV–134.5 eV, which were assigned the P2p doublet of BP, doublet P2p of P very low oxidized having the oxidation state P^3+^ and P2p doublet of P strong oxidized having the oxidized state P^5+^ [45]. The ratio between the intensities of the peaks at 129.99 eV and 133.63 eV is equal to 1.57.

Figure 9 shows the case of PDPA electrochemically functionalized BP sheets in: (i) XPS C1s, with the four peaks at 284.37 eV, 284.91 eV, 285.57 eV and 286.36 eV assigned to sp2 C atoms, the C-C and C-H bonds, the C-N bond and the C-O and C-N^+^ [42]; (ii) XPS N1s, with two peaks at 399.94 eV and 402.14 eV, assigned to the P=N bond [46,47,48] and protonated N (C-N^+^) [42]; (iii) XPS O1s, with two peaks at 532.04 eV and 533.38 eV, attributed to adsorbed molecular oxygen and the C-O bond; and (iv) XPS P2p, with four peaks at 134.06 eV–134.93 eV and 129.85 eV–130.71 eV, assigned to P=N bond and the P-P bond in BP [46,47,48]. The ratio between the intensities of the peaks at 129.99 eV and 133.63 eV is equal to 0.92. The smaller value of the ratio between the intensity of the peaks at 129.99 eV and 133.63 eV in the case of the PDPA electrochemically functionalized BP sheets in contrast with BP clearly confirms the decrease in the weight of P-P bonds and the increase in the weight of P=N bonds.

**Figure 10:**
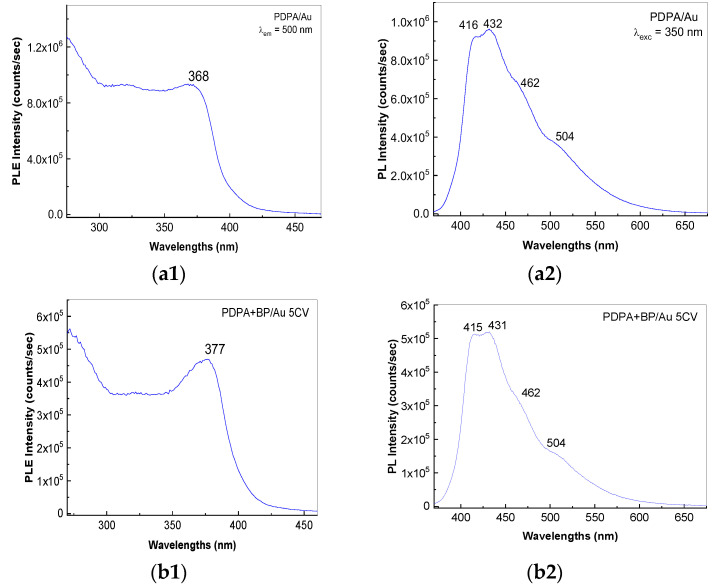

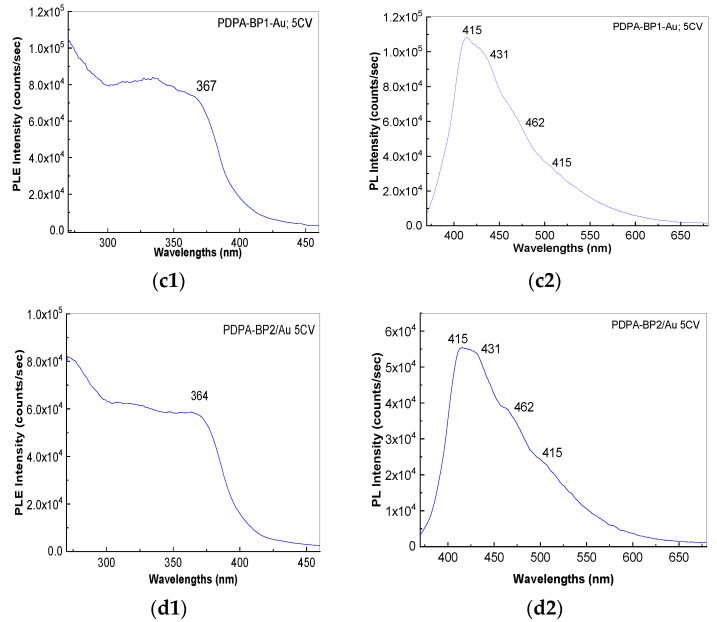
PLE and PL spectra of PDPA (**a1**,**a2**) and the DPA chemically functionalized and electrochemically oxidized BP sheets, with the synthesis mixture containing 0.1 mg/mL (**b1**,**b2**), 0.2 mg/mL (**c1**,**c2**) and 0.5 mg/mL (**d1**,**d2**) BP in DMF. PL spectra were recorded using an excitation wavelength equal to 350 nm. PLE spectra were recorded under an emission wavelength equal to 490 nm.

The electrochemical oxidation of DPA in the absence of the BP sheets, when the recording of the first five cyclic voltammograms on the Au electrode surface took place, led to films that are characterized by spectra of photoluminescence (PL (Figure 10(a_1_))) and photoluminescence excitation (PLE, Figure 10(a_2_)), having the following characteristics: (i) the PL spectrum of the sample of PDPA shows four emission bands with maxima at ~416 nm, 432 nm, 462 nm and 504 nm and (ii) the PLE spectrum highlights a band with the maximum at 368 nm. As the concentration of the BP sheets in the synthesis mixture increases, a gradual decrease in the intensity of the PL spectra of the macromolecular compound resulting from the electrochemical oxidation of the DPA chemically functionalized BP sheets was observed from 9.62 × 10^5^ counts/s (Figure 10(a_2_)) to 5.16 × 10^5^ counts/s (Figure 10(b_2_)), 1 × 10^5^ counts/s (Figure 10(c_2_)) and 5.5 × 10^4^ counts/sec (Figure 10(d_2_)). A decrease in the intensity of the PLE spectra was also observed in the case of Figure 10(a_1_–10d_1_). These variations are due to the electrochemical oxidation of the DPA chemically functionalized BP sheets, when are generated dimers, tetramers, oligomers and macromolecular compound. Taking into account the behavior of PL spectra, shown in Figure 10, these highlight the role of the BP sheets as a PL quenching agent of macromolecular compounds of DPA doped with PTA heteropolyanions.

**Figure 11:**
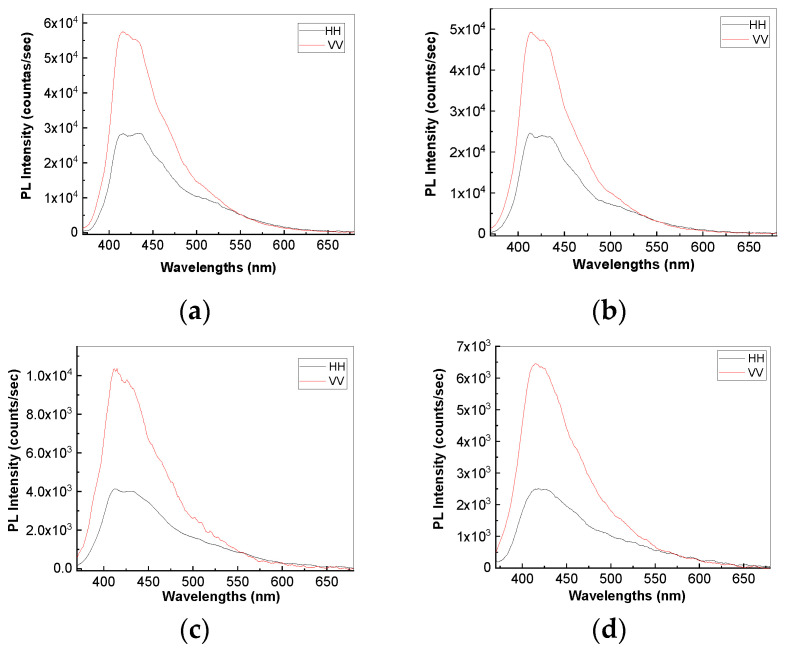
Anisotropic photoluminescence of PDPA (**a**) and composites resulting from the electrochemical oxidation of DPA chemically functionalized BP sheets, when the synthesis mixture contains the BP sheets in DMF having a concentration equal to 0.1 mg/mL (**b**), 0.2 mg/mL (**c**) and 0.5 mg/mL (**d**).

In order to understand the adsorption mode of macromolecular compounds of DPA doped with PTA heteropolyanions on the surface of the BP sheets, in Figure 11 are shown the PL spectra in polarized light of PDPA doped with the PTA heteropolyanions and of its composites based on macromolecular compounds of DPA and the BP sheets. To calculate the PL anisotropy, the following equation was used: r = (I_VV_ − (I_HV_/I_HH_) × I_VH_)/(I_VV_ + 2(I_HV_/I_HH_) × I_VH_) [39,49]. I_VV_ and I_HH_ correspond to the PL intensity when the excitation and emission polarizers are in the vertical and horizontal positions, respectively; I_VH_ and I_HV_ correspond to the PL intensity when the excitation and emission polarizers are in the vertical and horizontal positions and vice versa, respectively. The r values in the case of PDPA doped with PTA heteropolyanions and the composites of the type of the BP sheets electrochemically functionalized in the presence of DPA, when in the reaction mixture the concentrations of BP sheets are 0.1, 0.2 and 0.5 mg/mL, equal to 0.109, 0.103, 0.153 and 0.127, respectively. The wrapping angle of the BP sheets with macromolecular compounds of DPA—in other words, the adsorption angle of the polymer on the surface of the BP layers ((θ_PL_)—was calculated with the relation r = 0.4[3cos^2^θ_PL_ − 1)/2] [39,49], having values in the case of PDPA and the composites containing BP sheets equal to 44.1°, 44.7°, 42.4° and 39.9° when the concentrations of BP layers in the reaction mixture are 0, 0.1, 0.2 and 0.5 mg/mL, respectively. These values of the adsorption angle of the polymer on the surface of the BP layers indicate that the excitation and emission transition dipoles of macromolecular compounds are not parallel to the basal plane of the BP sheets.

## 4. Conclusions

In this work, new results are reported regarding the optical properties of the composite materials resulting from the electrochemical oxidation of the BP sheets chemically functionalized with DPA. The following conclusions can be drawn:

(i) The chemical interaction of the BP sheets with DPA involves a process of covalent functionalization that takes place with the formation of an iminophosphorene-type structure, which induces a decrease in the absorbance of the IR bands localized at 691–744 cm^−1^ and 876 cm^−1^. The formation of iminophosphorane groups on the BP surface was also reported through the interaction of black phosphorus with 4-benzoic acid azide [50].

(ii) The chemical interaction of the BP sheets having a P_x_O_y_ layer with PTA leads to a decrease in the oxidation state of the BP sheets, as a consequence of the interaction of P_x_O_y_ with water from H_3_PW_12_O_40_ x H_2_O.

(iii) The electrochemical oxidation of the DPA chemically functionalized BP sheets leads to the generation of dimers, tetramers and polymers when steric hindrance effects are highlighted by the increase in the absorbance of the IR bands peaking at 1022 cm^−1^ and 1072–1076 cm^−1^, attributed to vibrational modes of the deformation of the quinoid rings and stretching of the P-O-W bond; according to Raman spectroscopy studies, the composites resulting from the electrochemical oxidation of the DPA chemically functionalized BP sheets are characterized by an intense line at 1581 cm^−1^, which indicates a greater weight of quinoid rings compared to benzene rings in the macromolecular compound. The smaller ratio between the intensity of the XPS peaks at 129.99 eV and 133.63 eV in the case of the PDPA electrochemically functionalized BP sheets in contrast with BP clearly confirms the decrease in the weight of P-P bonds and the increase in the weight of P=N bonds.

(iv) The decrease in the intensity of the PL spectrum of DPA in the presence of the BP sheets highlights the role of the quenching agent of BP in the case of the PL spectra of DPA; the BP sheets play the role of a quenching agent of the PL spectra of the composites resulting from the electrochemical oxidation of the DPA chemically functionalized BP sheets in the presence of PTA.

(v) The value of the wrapping angle of the BP sheets with macromolecular compounds of DPA, when the concentrations of the BP layers in the reaction mixture are 0, 0.1, 0.2 and 0.5 mg/mL, ranging from 44.1° to 44.7°, 42.4° and 39.9°, respectively.

This study provides new opportunities for the use of BP sheets functionalized with PDPA doped with PTA heteropolyanions as an electrode active material in supercapacitor cells and rechargeable Li batteries as well as a flame-retardant agent. In this context, we point out that PDPA is one of the CPs that have been used for both supercapacitors and batteries (e.g., [51,52]), while in the case of BP, the applications in the field of energy storage have exploited the large size of the interlayer channel of 3.08 Å, which ensured a fast diffusion of ions [53]. Concerning the applications of BP as well as their composites as a flame-retardant agent, a recently study focused on BP functionalized with amine compounds for epoxy resin [20]. Risks that can lead to the fires and explosions of batteries as well as safety strategies have been reported in various reviews (e.g., [54]). One of the strategies adopted in it takes into account preventive measures by adding flame retardants in order to increase the thermal stability of the batteries. Considering this progress, in the particular case of PDPA-functionalized BP sheets, we anticipate that (i) the presence of BP sheets will facilitate an increase in ion diffusion, improving the discharge capacity of the supercapacitors and the specific capacity of the rechargeable Li batteries and (ii) an increase in the fire stability of rechargeable Li batteries, which use PDPA-functionalized BP sheets as the active electrode material, will occur.

## Data Availability

Not applicable.

## References

[1] Mack S.C., Witt H., Piro R.M., Gu L., Zuyderduyn S.D., Stutz A.M., Wang X., Gallo M., Garzia L., Zayne K. (2014). Epigenomic alterations define lethal CIMP-positive ependymomas of infancy. Nature.

[2] Brent J.R., Savjani N., Lewis E.A., Haigh S.J., Lewis D.J., O’Brien P. (2014). Production of few-layer phosphorene by liquid exfoliation of black phosphorus. Chem. Commun..

[3] Gui R., Jin H., Wang Z., Li J. (2018). Black phosphorus quantum dots: Synthesis, properties, functionalized modification and applications. Chem. Soc. Rev..

[4] Kou L., Chen C., Smith S.C. (2015). Phosphorene: Fabrication, Properties, and Applications. J. Phys. Chem. Lett..

[5] Kumar A. (2020). Controlled nanostructures and simultaneous passivation of black phosphorus (phosphorene) with Nafion. J. Mater. Res..

[6] Island J., Steele G.A., van der Zant H.S.J., Castellanos-Gomez A. (2015). Environmental instability of few-layer black phosphorus. 2D Mater..

[7] Favron A., Gaufrès E., Fossard F., Phaneuf-L’Heureux A.-L., Tang N.Y.-W., Levesque P., Loiseau A., Leonelli R., Francoeur S., Martel R. (2015). Photooxidation and quantum confinement effects in exfoliated black phosphorus. Nat. Mater..

[8] Woomer A.H., Farnsworth T.W., Hu J., Wells R.A., Donley C.L., Warren S.C. (2015). Phosphorene: Synthesis, Scale-Up, and Quantitative Optical Spectroscopy. ACS Nano.

[9] Yasaei P., Kumar B., Foroozan T., Wang C., Asadi M., Tuschel D., Indacochea J.E., Klie R.F., Salehi-Khojin A. (2015). High-Quality Black Phosphorus Atomic Layers by Liquid-Phase Exfoliation. Adv. Mater..

[10] Kang J., Wood J.D., Wells S.A., Lee J.-H., Liu X., Chen K.-S., Hersam M.C. (2015). Solvent Exfoliation of Electronic-Grade, Two-Dimensional Black Phosphorus. ACS Nano.

[11] Abellán G., Lloret V., Mundloch U., Marcia M., Neiss C., Görling A., Varela M., Hauke F., Hirsch A. (2016). Noncovalent Functionalization of Black Phosphorus. Angew. Chem. Int. Ed..

[12] Abellán G., Wild S., Lloret V., Scheuschner N., Gillen R., Mundloch U., Maultzsch J., Varela M., Hauke F., Hirsch A. (2017). Fundamental Insights into the Degradation and Stabilization of Thin Layer Black Phosphorus. J. Am. Chem. Soc..

[13] Zhao W., Xue Z., Wang J., Jiang J., Zhao X., Mu T. (2015). Large-Scale, Highly Efficient, and Green Liquid-Exfoliation of Black Phosphorus in Ionic Liquids. ACS Appl. Mater. Interfaces.

[14] Hirsch A., Hauke F. (2018). Post-Graphene 2D Chemistry: The Emerging Field of Molybdenum Disulfide and Black Phosphorus Functionalization. Angew. Chem. Int. Ed..

[15] Passaglia E., Cicogna F., Lorenzetti G., Legnaioli S., Caporali M., Serrano-Ruiz M., Ienco A., Peruzzini M. (2016). Novel polystyrene-based nanocomposites by phosphorene dispersion. RSC Adv..

[16] Zhang J., Ding W., Zhang Z., Xu J., Wen Y. (2016). Preparation of black phosphorus-PEDOT:PSS hybrid semiconductor composites with good film-forming properties and environmental stability in water containing oxygen. RSC Adv..

[17] Sun M.M., Wang W., Lv W., Lu M.X., Yan S.H., Liang L.Y., Ling Q.D. (2015). Synthesis of regular D-A1-D-A2 copolymers via direct arylation polycondensation and application in solar cells. Synth. Met..

[18] Fonsaca J., Domingues S.H., Orth E.S., Zarbin A.J.G. (2017). Air stable black phosphorous in polyaniline-based nanocomposite. Sci. Rep..

[19] Wu Z., Liang L., Zhu S., Guo Y., Yao Y., Yang Y., Gu S., Zhou Z. (2021). High Sensitivity of Ammonia Sensor through 2D Black Phosphorus/Polyaniline Nanocomposite. Nanomaterials.

[20] Liu S., Tao B., Zhao X., Chen G., Wang D.Y. (2021). Surface functionalization of black phosphorus via amine compounds and its impacts on the flame retardancy and thermal decomposition behaviors of epoxy resin. Polymers.

[21] Cao Y., Tian X., Gu J., Liu B., Zhang B., Song S., Fan F., Chen Y. (2018). Covalent Functionalization of Black Phosphorus with Conjugated Polymer for Information Storage. Angew. Chem. Int. Ed..

[22] Ren X., Mei Y., Lian P., Xie D., Yang Y., Wang Y., Wang Z. (2018). A Novel Application of Phosphorene as a Flame Retardant. Polymers.

[23] Luo M., Cheng W., Zeng X., Mei L., Liu G., Deng W. (2019). Folic Acid-Functionalized Black Phosphorus Quantum Dots for Targeted Chemo-Photothermal Combination Cancer Therapy. Pharmaceutics.

[24] Wang D., Guo G.-C., Wei X.-L., Liu L.-M., Zhao S.-J. (2016). Phosphorene ribbons as anode materials with superhigh rate and large capacity for Li-ion batteries. J. Power Sources.

[25] Luo W., Shen F., Bommier C., Zhu H., Ji X., Hu L. (2016). Na-Ion Battery Anodes: Materials and Electrochemistry. Accounts Chem. Res..

[26] Rahman M.Z., Kwong C.W., Davey K., Qiao S.Z. (2016). 2D phosphorene as a water splitting photocatalyst: Fundamentals to applications. Energy Environ. Sci..

[27] Cui S., Pu H., Wells S.A., Wen Z., Mao S., Chang J., Hersam M.C., Chen J. (2015). Ultrahigh sensitivity and layer-dependent sensing performance of phosphorene-based gas sensors. Nat. Commun..

[28] Lee J., Seo J., Oh J.H., Shin M. (2016). Nonorthogonal tight-binding parameterization of single-layer phosphorene under biaxial strain and application to FETs. Nanotechnology.

[29] Smaranda I., Benito A.M., Maser W.K., Baltog I., Baibarac M. (2014). Electrochemical Grafting of Reduced Graphene Oxide with Polydiphenylamine Doped with Heteropolyanions and Its Optical Properties. J. Phys. Chem. C.

[30] Guo Y., Li K., Yu X., Clark J.H. (2008). Mesoporous H3PW12O40-silica composite: Efficient and reusable solid acid catalyst for the synthesis of diphenolic acid from levulinic acid. Appl. Catal. B Environ..

[31] Legagneux N., Basset J.-M., Thomas A., Lefebvre F., Goguet A., Sá J., Hardacre C. (2009). Characterization of silica-supported dodecatungstic heteropolyacids as a function of their dehydroxylation temperature. Dalton Trans..

[32] Kim J., Lee J.-U., Lee J., Park H.J., Lee Z., Lee C., Cheong H. (2015). Anomalous polarization dependence of Raman scattering and crystallographic orientation of black phosphorus. Nanoscale.

[33] Mitrović A., Wild S., Lloret V., Fickert M., Assebban M., Márkus B.G., Simon F., Hauke F., Abellán G., Hirsch A. (2020). Interface Amorphization of Two-Dimensional Black Phosphorus upon Treatment with Diazonium Salts. Chem. A Eur. J..

[34] Edmonds M.T., Tadich A., Carvalho A., Ziletti A., O’Donnell K.M., Koenig S.P., Coker D.F., Özyilmaz B., Neto A.H.C., Fuhrer M.S. (2015). Creating a Stable Oxide at the Surface of Black Phosphorus. ACS Appl. Mater. Interfaces.

[35] Pires L.H., de Oliveira A.N., Monteiro O.V., Angélica R.S., Costa C., Zamian J.R., Nascimento L.D., Filho G.N.D.R. (2014). Esterification of a waste produced from the palm oil industry over 12-tungstophosforic acid supported on kaolin waste and mesoporous materials. Appl. Catal. B Environ..

[36] Essayem N., Holmqvist A., Gayraud P., Vedrine J., Ben Taarit Y. (2001). In Situ FTIR Studies of the Protonic Sites of H3PW12O40 and Its Acidic Cesium Salts MxH3−xPW12O40. J. Catal..

[37] Quillard S., Louarn G., Lefrant S., Macdiarmid A.G. (1994). Vibrational analysis of polyaniline: A comparative study of leucoemeraldine, emeraldine, and pernigraniline bases. Phys. Rev. B.

[38] Baibarac M.A., Radu A., Cristea M., Cercel R., Smaranda I. (2020). UV Light Effect on Cationic Photopolymerization of the SU8 Photoresist and Its Composites with Carbon Nanotubes: New Evidence Shown by Photoluminescence Studies. J. Phys. Chem. C.

[39] Kuo C.-T., Chiou W.-H. (1997). Field-effect transistor with polyaniline thin film as semiconductor. Synth. Met..

[40] de Santana H., Temperini M.L.A., Rubim J. (1993). In situ resonance Raman and reflectance spectroscopic study of the electrochemical oxidation of diphenylamine. J. Electroanal. Chem..

[41] Fei R., Yang L. (2014). Lattice vibrational modes and Raman scattering spectra of strained phosphorene. Appl. Phys. Lett..

[42] Wu M.-S., Wen T.-C., Gopalan A. (2001). Electrochemical Copolymerization of Diphenylamine and Anthranilic Acid with Various Feed Ratios. J. Electrochem. Soc..

[43] Nadaf S.N., Patil S.S., Kalantre V.A., Mali S.S., Hong C.K., Mane S.R., Bhosale P.M. (2021). Optimization and com-parative analysis of Cs ion intercalated H3PMo12O40 photocathode: One-step hydrothermal strategy. J. Mater. Sci. Mater. Electron..

[44] Chen C., Peng J., Shen Y., Chen D., Zhang H., Meng C. (2011). Fabrication and Characterization of LuminescentMul-tilayer Films Based on Polyoxometalates and Fuchsin Basic. Z. Nat. B.

[45] Luo W., Zemlyanov D.Y., Milligan C.A., Du Y., Yang L., Wu Y., Ye P.D. (2016). Surface chemistry of black phosphorus under a controlled oxidative environment. Nanotechnology.

[46] Luaña V., Pendás A.A.M., Costales A., And G.A.C., García-Alonso F.J. (2001). Topological Analysis of Chemical Bonding in Cyclophosphazenes. J. Phys. Chem. A.

[47] Vassileva P., Krastev V., Lakov L., Peshev O. (2004). XPS determination of the binding energies of phosphorus and nitrogen in phosphazenes. J. Mater. Sci..

[48] Dake L., Baer D., Ferris K., Friedrich D. (1990). Ligand and structure effects on the N-P bonds of phosphazenes. J. Electron. Spectrosc. Relat. Phenom..

[49] Shea M.J., Mehlenbacher R.D., Zanni M.T., Arnold M.S. (2014). Experimental Measurement of the Binding Configuration and Coverage of Chirality-Sorting Polyfluorenes on Carbon Nanotubes. J. Phys. Chem. Lett..

[50] Liu Y., Gao P., Zhang T., Zhu X., Zhang M., Chen M., Du P., Wang G., Ji H., Yang J. (2018). Azide Passivation of Black Phosphorus Nanosheets: Covalent Functionalization Affords Ambient Stability Enhancement. Angew. Chem. Int. Ed..

[51] Baibarac M., Baltog I., Lefrant S., Gomez-Romero P. (2011). Polydiphenylamine/carbon nanotube composites for applications in rechargeable lithium batteries. Mater. Sci. Eng. B.

[52] Baibarac M., Baltog I., Frunza S., Magrez A., Schur D., Zaginaichenko S.Y. (2013). Single-walled carbon nanotubes functionalized with polydiphenylamine as active materials for applications in the supercapacitors field. Diam. Rel. Mater..

[53] Zhang H., Wang X., Ma H., Xue M. (2021). Recent Progresses on Applications of Conducting Polymers for Modifying Electrode of Rechargeable Batteries. Adv. Energy Sustain. Res..

[54] Kong L., Li C., Jiang J., Pecht M.G. (2018). Li-Ion Battery Fire Hazards and Safety Strategies. Energies.

